# Disruption of KCC2 in Parvalbumin-Positive Interneurons Is Associated With a Decreased Seizure Threshold and a Progressive Loss of Parvalbumin-Positive Interneurons

**DOI:** 10.3389/fnmol.2021.807090

**Published:** 2022-02-03

**Authors:** Tanja Herrmann, Melanie Gerth, Ralf Dittmann, Daniel Pensold, Martin Ungelenk, Lutz Liebmann, Christian A. Hübner

**Affiliations:** Institute of Human Genetics, University Hospital Jena, Jena, Germany

**Keywords:** KCC2, GABA, interneuron, epilepsy, inhibition

## Abstract

GABA_A_ receptors are ligand-gated ion channels, which are predominantly permeable for chloride. The neuronal K-Cl cotransporter KCC2 lowers the intraneuronal chloride concentration and thus plays an important role for GABA signaling. KCC2 loss-of-function is associated with seizures and epilepsy. Here, we show that KCC2 is expressed in the majority of parvalbumin-positive interneurons (PV-INs) of the mouse brain. PV-INs receive excitatory input from principle cells and in turn control principle cell activity by perisomatic inhibition and inhibitory input from other interneurons. Upon Cre-mediated disruption of KCC2 in mice, the polarity of the GABA response of PV-INs changed from hyperpolarization to depolarization for the majority of PV-INs. Reduced excitatory postsynaptic potential-spike (E-S) coupling and increased spontaneous inhibitory postsynaptic current (sIPSC) frequencies further suggest that PV-INs are disinhibited upon disruption of KCC2. *In vivo*, PV-IN-specific KCC2 knockout mice display a reduced seizure threshold and develop spontaneous sometimes fatal seizures. We further found a time dependent loss of PV-INs, which was preceded by an up-regulation of pro-apoptotic genes upon disruption of KCC2.

## Introduction

Brain function depends on highly interconnected networks of excitatory pyramidal neurons and inhibitory interneurons. The latter are predominantly locally projecting neurons, which release GABA to refine and shape circuit output by modulating the gain, timing, tuning, bursting properties of pyramidal cell firing, and selective filtering of synaptic excitation (Roux and Buzsaki, 2015). GABAergic interneurons account for roughly 20-30% of the overall neuronal population in the mammalian cerebral cortex (Hendry et al., 1987) and can be classified by different features. The most common classification is based on the expression of different molecular markers such as parvalbumin (PV), calbindin, somatostatin, and others (Markram et al., 2004). PV-positive interneurons (PV-INs) are the most common subtype (Celio, 1986) and are characterized by a fast-spiking phenotype, low input resistance and high-amplitude rapid after-hyperpolarization (Hu et al., 2014). They can be further divided into basket cells, which innervate the soma and proximal dendrites, and chandelier cells that synapse onto the axon initial segment. GABAergic interneuron subtypes not only target different domains of pyramidal cells but also other interneurons (Gibson et al., 1999; Blatow et al., 2003; Hu et al., 2011; Jiang et al., 2013; Pfeffer et al., 2013). If interneuron function is impaired, this can affect higher brain functions and may result in seizures (Liu et al., 2014).

The polarity of the response to GABA critically depends on the intraneuronal chloride concentration. The intraneuronal chloride concentration [Cl^–^]_i_ is mainly determined by the interplay between the Na^+^-dependent KCl-cotransporter NKCC1, which uses the Na^+^ gradient to raise [Cl^–^]_i_, and the Na^+^-independent KCl-cotransporter KCC2, which uses the K^+^-gradient to lower [Cl^–^]_i_ (Payne et al., 1996, 2003; Blaesse et al., 2009; Kilb, 2012; Luhmann et al., 2014; Virtanen et al., 2020). The GABA-mediated hyperpolarizing chloride current is typically established during the first postnatal weeks in rodents (Rivera et al., 1999; Hubner et al., 2001; Ben-Ari et al., 2012). Mice with a total deletion of *Slc12a5* (*Kcc2*) die immediately after birth because of a failure to breathe (Hubner et al., 2001), while hypomorphic mice, which express about 5–8% of wild-type KCC2 protein levels, exhibit spontaneous generalized seizures and die before the third postnatal week (Woo et al., 2002). Notably, KCC2 loss-of-function mutations in humans are associated with inherited febrile seizures, severe genetic generalized epilepsy and epilepsy of infancy with migrating focal seizures (Hubner, 2014; Kahle et al., 2014; Puskarjov et al., 2014; Kahle et al., 2016; Saitsu et al., 2016; Di Cristo et al., 2018).

Here, we addressed the role of KCC2 in PV-INs. We show that the targeted disruption of KCC2 under control of the PV-promoter in mice results in a reduced seizure threshold with progressive loss of PV-INs. *In vitro*, the response of PV-INs to GABA was converted from hyperpolarizing to depolarizing. In agreement, network dependent GABA release was increased. The transcriptional profiling of PV-INs isolated from these mice suggest that stress related pathway may trigger apoptosis.

## Results

### KCC2 Is Broadly Expressed in PV-Positive Neurons

**FIGURE 1:**
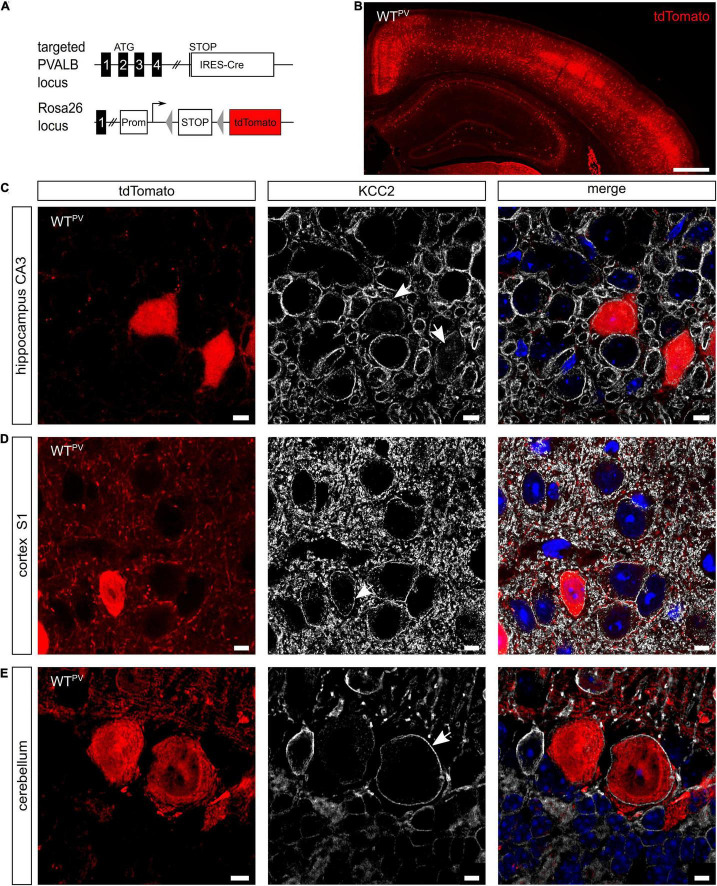
KCC2 is broadly expressed in PV-INs. **(A)** To label PV-INs we mated a mouse line expressing Cre-recombinase under the control of the PV promoter (PV-Cre) (Hippenmeyer et al., 2005) with a tdTomato reporter line (Madisen et al., 2010). Cre-mediated excision of the STOP cassette results in the expression of tdTomato. **(B)** Coronal section of the cortex and the hippocampus of an 8-week-old mouse transgenic for Cre-recombinase and the tdTomato allele (WT^PV^). Scale bars 500 μm. **(C–E)** Brain sections from WT^PV^ mice were stained for KCC2. tdTomato-positive neurons in the hippocampus **(C)**, somatosensory cortex **(D)**, and cerebellum **(E)** displayed a clear KCC2 signal at the plasma membrane (arrows). Nuclei were counterstained with DAPI. Scale bars 10 μm.

PV-INs constitute ∼2.6% of overall neurons in the CA1 region of the hippocampus and ∼40% of GABAergic neocortical neurons (Rudy et al., 2011). To visualize PV-expressing cells we mated mice expressing Cre-recombinase under control of the PV promoter (PV-Cre) (Hippenmeyer et al., 2005) with a tdTomato reporter line (tdTomato) (Madisen et al., 2010) to obtain mice expressing tdTomato in PV-INs (WT^PV^ mice) (Figures 1A,B). In brain sections from 8-week-old WT^PV^ mice nearly all cells expressing tdTomato also stained for PV (Supplementary Figure 1A). When we stained such brain sections for KCC2, we observed that the vast majority of tdTomato-positive neurons also stained for KCC2 (75.46 ± 2.412%). This included PV-INs of the hippocampus (Figure 1C), the somatosensory cortex (Figure 1D) as well as Purkinje cells in the cerebellum (Figure 1E).

These data confirm that the majority of PV-positive neurons express KCC2.

### Disruption of KCC2 Under the Control of the PV-promoter Increases Seizure Susceptibility

**FIGURE 2:**
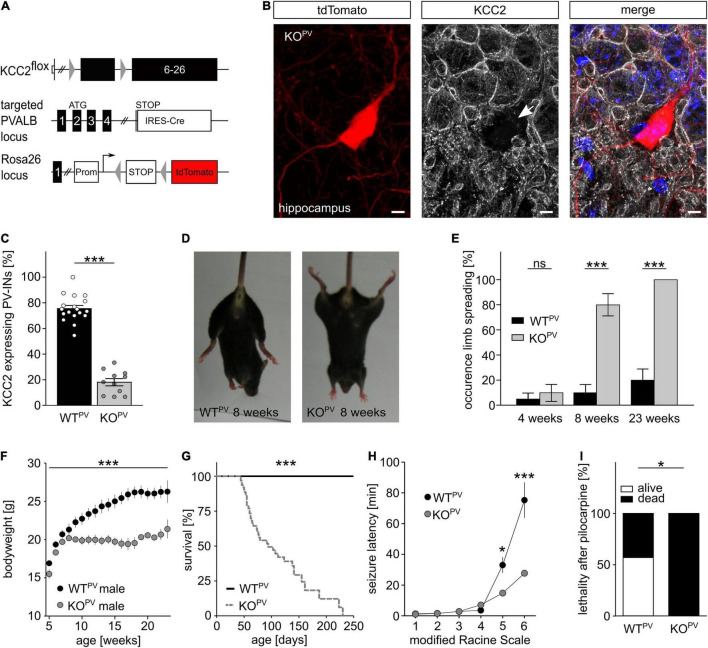
Fatal epileptic activity upon disruption of KCC2 in PV-INs. **(A–C)** We mated our floxed *Kcc2* line (*Kcc2*^flox/flox^) (Seja et al., 2012) with WT^PV^ mice to delete exons 2-5 of the *KCC2* gene **(A)**. The KCC2-labeled section of an 8-week-old *KCC2*^flox/flox^/PV-Cre/tdTomato (KCC2 KO^PV^) mouse shows that the disruption of KCC2 was effective in the majority of tdTomato-labeled interneurons (arrow) **(B)**. Scale bars 5 μm. The quantification of KCC2-labeled tdTomato-positive neurons in cortex of WT^PV^ and KCC2 KO^PV^ mice confirms the deletion of KCC2 in most PV-INs at 8 weeks of age **(C)**. The ratio of tdTomato-positive with a clear plasma membrane labeling for KCC2 and the total number of tdTomato-positive cells was determined for the somatosentory cortex (WT: 75.5 ± 2.4%; KO: 18.1 ± 2.8%; quantification from *n* = 9 sections and *N* = 3 mice; Student’s unpaired *t*-test; ***p < 0.001). **(D,E)** KCC2 KO^PV^ mice spread their hind limbs **(D)**. Quantification of hind limb spreading in WT^PV^ and KCC2 KO^PV^ mice at different ages (*N* = 20/20 mice, Kruskal-Wallis Test; *post hoc* Dunn’s multiple comparison; ns not significant; ***p < 0.001). **(F)** The gain in body weight is decreased in KCC2 KO^PV^ mice (*N* = 10/9 mice; 2-way ANOVA; Bonferroni post-test; ***p < 0.0001). **(G)** KCC2 KO^PV^ animals have a shortened life span with a mean survival of 94 days (*N* = 66/44 mice; Mantel-Cox Test; ***p < 0.0001). **(H,I)** 8-week-old WT^PV^ and KCC2 KO^PV^ mice were challenged with pilocarpine after LiCl sensitization to induce epileptic seizures. All challenged animals developed generalized tonic-clonic seizures. The seizure threshold was decreased in KCC2 KO^PV^ mice compared to WT^PV^ mice (*N* = 6/6 mice; 2-way ANOVA; Bonferroni post-test; ***p = 0.0003) and the seizure related lethality increased (I) (*N* = 6/6; 2-way ANOVA; Bonferroni post-test; *p = 0.037).

Previously, we reported that Cre-recombinase mediated deletion of exons 2-5 of the floxed *Kcc2* allele (*Kcc2*^flox/flox^) results in a *Kcc2* knockout (KO) allele (Seja et al., 2012). To disrupt KCC2 in PV-INs we mated the floxed *Kcc2* line with the WT^PV^ line (Hippenmeyer et al., 2005), which also carried the tdTomato transgene (Figure 2A). Cre-positive mice carrying one floxed *Kcc2* allele did not show any obvious abnormalities (data not shown). Homozygous *Kcc2*^flox/flox^/PV-Cre/tdTomato (KCC2 KO^PV^) mice were born from heterozygous matings at the expected ratio. Staining of brain sections dissected from 8-week-old mice (Figure 2B) revealed that roughly 80% of the tdTomato-labeled cells in somatosensory cortex showed robust labeling for KCC2 in WT^PV^ mice, while only less than 20% of the tdTomato-positive cells labeled for KCC2 in KCC2 KO^PV^ mice (Figure 2C). Similar results were obtained for the hippocampus (Supplementary Figures 1B,C).

At 8 weeks of age almost 80% of the KCC2 KO^PV^ mice spread their hind-limbs when lifted by the tail, which was a very rare finding in control mice (Figures 2D,E). Fifteen weeks later, the hind-limb spreading was a consistent finding in KCC2 KO^PV^ mice. The Rotarod analysis showed significant motor impairments in KCC2 KO^PV^ mice, which started between 6 and 8 weeks of age (Supplementary Figure 2A). Because of the progressive motor impairments, we assessed mice in a fear conditioning paradigm, which is largely independent of motor functions. While both genotypes remembered the conditioned stimulus and the context of the aversive stimulus, KCC2 KO^PV^ mice displayed an increased anxiety-like behavior (Supplementary Figure 2B). In agreement, systemic corticosterone levels were increased in 8-week-old KCC2 KO^PV^ mice (Supplementary Figure 2C).

Notably, KCC2 KO^PV^ mice also showed a delayed increase in bodyweight from 8 weeks of age onward (Figure 2F) and the lethality drastically increased with almost no KCC2 KO^PV^ mouse surviving beyond 8 months of age (Figure 2G).

Repetitively, animal caretakers reported spontaneous seizures in KCC2 KO^PV^ mice, which were first noticed at 8 weeks of age with an age-dependent increase of such events. Some seizures ended fatally, thus at least in part explaining the high lethality of KCC2 KO^PV^ mice.

To assess the seizure susceptibility in a quantitative manner, we challenged 8-week-old KCC2 KO^PV^ and control mice with 80 mg/kg bodyweight pilocarpine after priming with 423 mg/kg lithium chloride i.p. 18 h before the challenge (Lodato et al., 2011). Latencies for the loss of postural control and the onset of generalized tonic-clonic seizures were drastically reduced in KCC2 KO^PV^ mice (Figure 2H). While the duration of Racine levels 4 and 5 was significantly shortened, the duration of Racine level 3 was prolonged (Supplementary Figures 2D,E). Moreover, the pilocarpine-induced seizures were always lethal in KCC2 KO^PV^ mice, while more than half of the control mice survived pilocarpine-induced seizures (Figure 2I).

Taken together, the disruption of KCC2 under control of the PV-promoter increases the seizure susceptibility.

### KCC2 Controls the Polarity of GABAergic Responses in PV-Positive Interneurons

**FIGURE 3:**
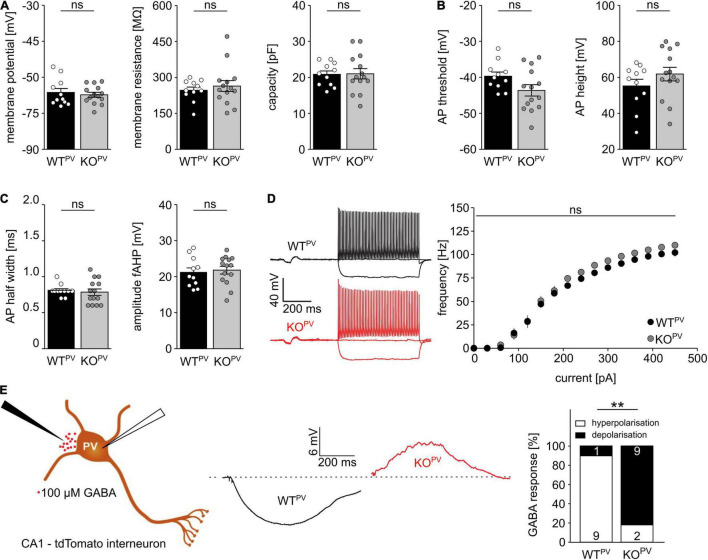
Disruption of KCC2 does not change the intrinsic excitability of PV-INs but changes the polarity of their GABA response. **(A)** Intrinsic properties such as resting membrane potential, membrane resistance and capacity are not altered in the absence of KCC2 (*n* = 11/14 cells from *N* = 5/6 mice; unpaired Student’s *t*-test; ns not significant). **(B)** Action potential (AP) threshold and amplitude are as well unchanged in the absence of KCC2 (*n* = 11/14 cells from *N* = 5/6 mice; unpaired Student’s *t*-test; ns not significant). **(C)** Action potential half width (left) and fast after-hyperpolarization (fAHP) (right) of PV-INs is similar in WT^PV^ and KO^PV^ (*n* = 11/14 cells from *N* = 5/6 mice; unpaired Student’s *t*-test; ns not significant). **(D)** The spike frequency upon current injection is not altered by disruption of KCC2 (*n* = 11/14 cells from *N* = 5/6 mice; 2-way ANOVA; Bonferroni post-test; ns not significant). **(E)** 100 mM GABA puffs were applied to tight seal (> 4 GΩ) patched tdTomato-labeled interneurons in the CA1 region of acute brain sections of 8-week-old WT^PV^ and KCC2 KO^PV^ mice (*n* = 10/11 cells from *N* = 5/6 mice; Fisher’s exact test; **p = 0.0019).

Resting membrane potential and basic passive membrane properties like membrane resistance and capacity were not altered in PV-INs of 8-week-old KCC2 KO^PV^ mice (Figure 3A). Neither the action potential threshold and height (Figure 3B) nor action potential half-width and amplitudes of fast after-hyperpolarizations (fAHP) differed between genotypes (Figure 3C). Also the action potential frequency in response to current injections did not differ between genotypes (Figure 3D). Thus, the basic excitability of PV-INs was not altered upon disruption of KCC2.

To scrutinize the role of KCC2 for the regulation of the intracellular chloride concentration and thus GABA signaling we used a tight cell patch approach without affecting transmembrane ionic gradients (Perkins, 2006) and tested the polarity of GABA responses in tdTomato labeled interneurons in CA1 of acute brain slices dissected from 8-week-old WT^PV^ mice. Consistent with a role of KCC2 for GABA responses in PV-positive interneurons, 9 from 10 cells in acute brain slices of control mice displayed a hyperpolarizing response, while it was depolarizing in 9 out of 11 cells for KCC2 KO^PV^ mice (Figure 3E).

**FIGURE 4:**
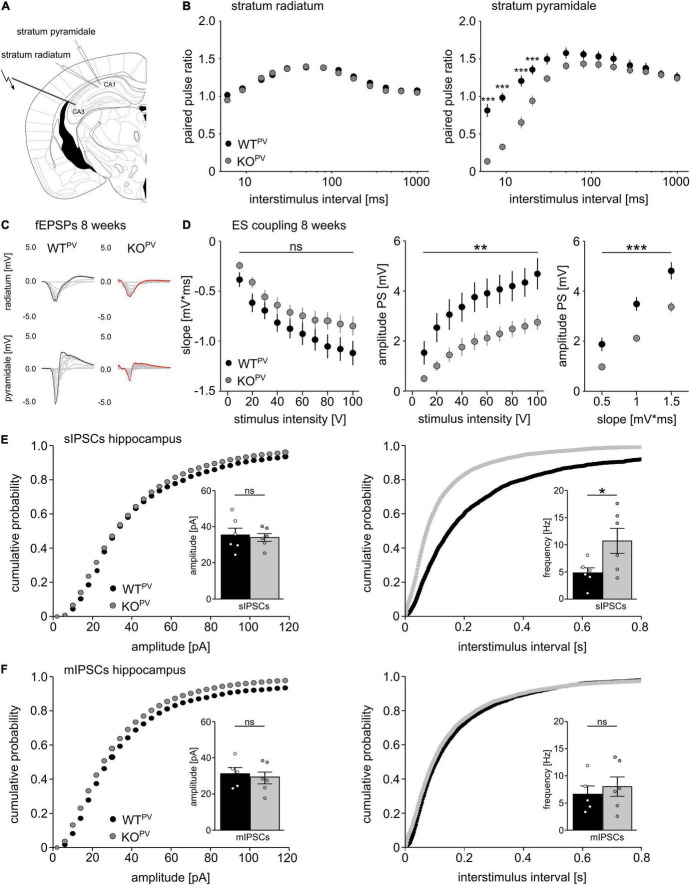
Disinhibition of PV-INs upon disruption of KCC2. **(A)** Position of stimulus and recording electrodes for field recordings in the *stratum pyramidale* and *radiatum* of CA1. **(B)** Paired-pulse ratios are reduced in the *stratum pyramidale* (*n* = 28/24 cells from *N* = 9/9 mice), but not in the *stratum radiatum* (*n* = 35/34 cells from *N* = 11/11 mice) of KCC2 KO^PV^ mice (2-way ANOVA repeated measure; Bonferroni post-test; ***p < 0.0001). **(C)** Representative examples of fESPs recorded in the *stratum radiatum* and *stratum pyramidale* in KCC2 WT^PV^ and KCC2 KO^PV^ mice. **(D)** E-S-coupling is diminished in 8-week-old KCC2 KO^PV^ mice (*n* = 14/17 slices from *N* = 4/4 mice, 2-way ANOVA repeated measure; Bonferroni post-test; **p = 0.008; ***p < 0.0001). **(E)** The frequency, but not the amplitude of spontaneous inhibitory post synaptic currents (sIPSCs) is increased in CA1 pyramidal neurons in KCC2 KO^PV^ mice (*N* = 6/6 mice, unpaired Student’s *t*-test; Kolmogorov-Smirnov test; ns not significant; *p = 0.0398). **(F)** Miniature inhibitory postsynaptic currents (mIPSCs) upon tetrodotoxin (TTX) inhibition of spontaneous network driven events are not changed in 8-week-old KCC2 KO^PV^ mice (*N* = 6/6 mice; unpaired Student’s *t*-test; Kolmogorov-Smirnov test; ns not significant).

We used field recordings to assess the consequences of the disruption of KCC2 in PV-INs for network excitability (Figure 4A). While the paired-pulse ratio was not changed in the *stratum radiatum* (Figure 4B), it was diminished for short interstimulus intervals in the *stratum pyramidale* of CA1 in 8-week-old KCC2 KO^PV^ mice (Figure 4C). This is compatible with increased GABAergic inhibition, because the response to the second stimulus at short intervals is limited via GABA_A_ dependent feed forward inhibition, while for intervals between 100–125 ms activation of presynaptic GABA_B_ autoreceptor activation dominates (Davies et al., 1990; Steffensen and Henriksen, 1991).

While slopes of extracellular recordings of field excitatory postsynaptic potentials (fEPSPs) recorded in the *stratum pyramidale* in response to a single stimulation of Schaffer collaterals did not differ between genotypes, amplitudes of population spikes and E-S coupling were decreased in KCC2 KO^PV^ mice (Figure 4D).

Next, we patched CA1 pyramidal cells and measured spontaneous GABA release in acute slices of 8-week-old control and KCC2 KO^PV^ mice. The frequency of spontaneous inhibitory postsynaptic currents (sIPSCs) was clearly increased in KCC2 KO^PV^ mice (Figure 4E). When we blocked spontaneous action potentials with tetrodotoxin (TTX), miniature inhibitory postsynaptic currents (mIPSCs) did not differ between genotypes (Figure 4F). Similar results were obtained for the motor cortex and the somatosensory cortex (Supplementary Figure 3).

Taken together, these data suggest that PV-INs are disinhibited in the absence of KCC2.

### Progressive Loss of PV-Positive Interneurons Upon Disruption of KCC2

**FIGURE 5:**
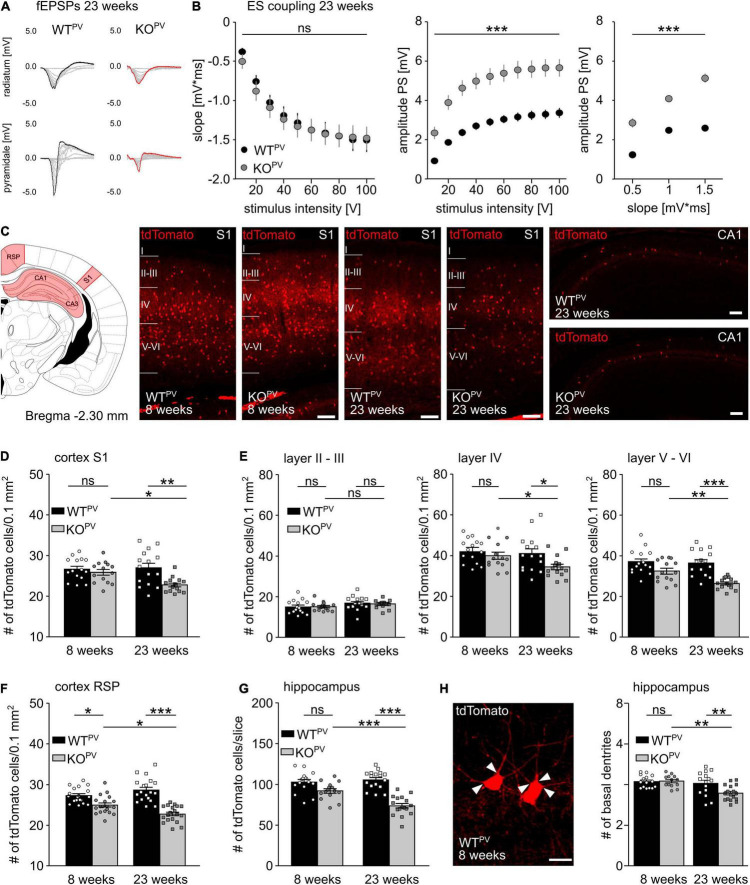
Loss of PV-INs in KCC2 KO^PV^ mice. **(A)** Representative examples of fEPSPs recorded in the *stratum radiatum* and *stratum pyramidale* of 23-week-old WT^PV^ and KCC2 KO^PV^ mice. **(B)** E-S-coupling is increased in 23-week-old KCC2 KO^PV^ mice (*n* = 24/33 slices from *N* = 5/5 mice; 2-way ANOVA repeated measure; Bonferroni post-test; ***p < 0.0001). **(C)** Illustration of the brain regions (red) for the quantification of tdTomato-positive neurons. Representative images of tdTomato-positive cells from the somatosensory cortex (S1) of WT^PV^ (left) and KCC2 KO^PV^ (right) mice at 23 weeks of age and hippocampus CA1 of 23-week-old mice. Scale bars 100 μm. **(D)** Number of tdTomato-positive cells per 0.1 mm^2^ of a 30 μm thick section of S1 of 8- and 23-week-old KCC2 WT^PV^ and KO^PV^ mice (*n* = 15/15 slices; *N* = 5/5 mice; 1-way ANOVA; Bonferroni post-test; *p < 0.05; **p < 0.01). **(E)** Number of tdTomato-positive cells per 0.1 mm^2^ and layer of a 30 μm thick section of S1 (*n* = 15/15 slices; *N* = 5/5 mice; 1-way ANOVA; Bonferroni post-test; ns not significant; *p < 0.05; **p < 0.01; ***p < 0.001). **(F)** Number of tdTomato-positive cells per 0.1mm^2^ of a 30 μm thick section of the retrosplenial cortex (RSP) of 8- and 23-week-old WT^PV^ and KCC2 KO^PV^ mice (*n* = 17/15 slices; *N* = 5/5 mice; 1-way ANOVA; Bonferroni post-test; *p < 0.05; ***p < 0.0001). **(G)** Quantification of tdTomato-positive cells in a 30 μm thick hippocampal section (Bregma -2.3 mm, 1 hemisphere) of 8- and 23-week-old WT^PV^ and KCC2 KO^PV^ mice (*n* = 15/15 slices; *N* = 5/5 mice; 1-way ANOVA; Bonferroni post-test; ***p < 0.0001). Individual numbers for D-G are provided in Supplementary Table 1. **(H)** The number of basal dendrites (arrowheads) of tdTomato-positive neurons in the hippocampus is slightly decreased in 23-week-old KCC2 KO^PV^ mice (WT 8 weeks: 4.2 ± 0.1; KO 8 weeks: 4.2 ± 0.1; WT 23 weeks: 4.1 ± 0.2; KO 23 weeks: 3.6 ± 0.1; *n* = 15/15 slices from *N* = 5/5 mice; 1-way ANOVA; Bonferroni‘s multiple comparison test; **p < 0,001).

When we re-assessed E-S coupling in the CA1 of acute brain slices obtained from 23-week-old mice, E-S coupling was increased in KCC2 KO^PV^ compared to WT^PV^ mice (Figures 5A,B). This finding suggested that GABAergic inhibition may be compromised in older KCC2 KO^PV^ mice. We hence wondered whether loss of KCC2 may affect the long-term maintenance of PV-INs. Therefore, we counted the number of tdTomato-labeled interneurons in the cortex and the hippocampus of 8- and 23-week-old control and KCC2 KO^PV^ mice (Figure 5C). In the somatosensory cortex (S1) we found no difference in the number of tdTomato-labeled interneurons at 8 weeks of age, while the number was clearly reduced in 23-week-old KCC2 KO^PV^ mice (Figures 5D,E). In the retrosplenial cortex (RSP), we observed a slight decrease already at 8 weeks of age, which was even more pronounced at 23 weeks (Figure 5F). A loss of PV-INs was also evident in the hippocampus of KCC2 KO^PV^ mice at 23 weeks of age (Figure 5G). The remaining PV-INs in CA1 also showed some structural abnormalities as evidenced by less basal dendrites compared to WT^PV^ mice (Figure 5H). Overall, the architecture of neurites appeared to be altered in older KCC2 KO^PV^ mice (Supplementary Figure 4).

### Perineuronal Networks Decrease in KCC2 KO^PV^ Mice

**FIGURE 6:**
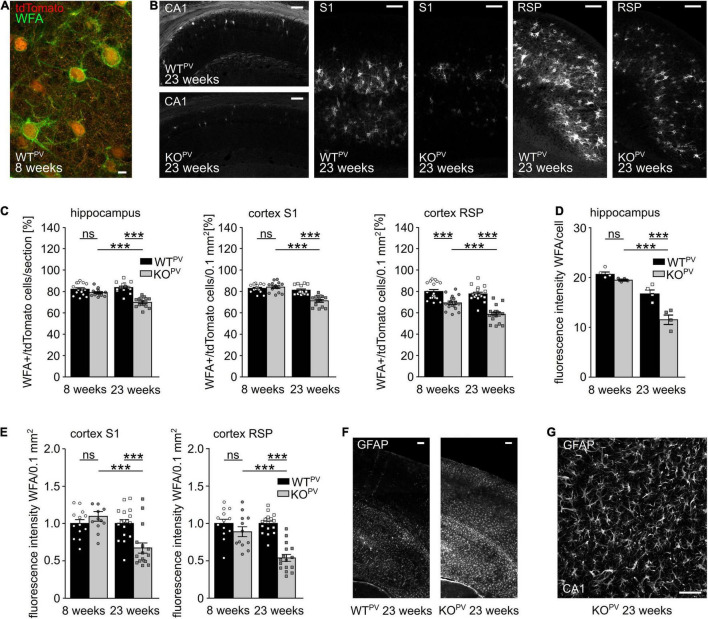
Perineuronal networks (PNN) decrease in KCC2 KO^PV^ mice. **(A)** Representative WFA stainings of the somatosensory cortex of a WT^PV^ mouse at the age of 8 weeks. Scale bar 5 μm. **(B)** Representative WFA stainings of the hippocampus (left), the S1 (middle) and RSP cortex (right) of WT^PV^ and KCC2 KO^PV^ mice at 23 weeks of age. Scale bars 100 μm. **(C)** Quantification of WFA^+^/tdTomato cells in the hippocampus (left), S1 (middle), and RSP (right) cortex of WT^PV^ and KCC2 KO^PV^ mice at the age of 8 and 23 weeks shows a reduced proportion of ensheathed PV-INs in older KO^PV^ mice (*n* = 15/15 slices, *N* = 5/5 mice; 1-way ANOVA; Bonferroni’s multiple comparison test; ns not significant; ***p < 0.001). **(D,E)** WFA signal intensity per individual cell in the hippocampus (D) (*n* = 12/9 slices; *N* = 4/3 mice; 1-way ANOVA; Bonferroni post-test; ns not significant; ***p < 0.0001). WFA signal intensity per 0.1 mm^2^ in the S1 cortex (left) and RSP (right) decreases over time in KCC2 KO^PV^ mice (*n* = 13/15 slices; *N* = 5/5 mice; 1-way ANOVA Bonferroni post-test; ns not significant; ***p < 0.0001). **(F)** Representative GFAP staining of the cortex and hippocampus of WT^PV^ and KCC2 KO^PV^ at the age of 23 weeks. Scale bar 100 μm. **(G)** Close up of GFAP -stained reactive astrocytes in CA3 in KCC2 KO^PV^. Scale bar 50 μm.

Many PV-INs are ensheathed by perineuronal nets (PNN) (Wen et al., 2018; Carceller et al., 2020; Figure 6A), extracellular matrix (ECM) assemblies that can be easily detected by lectins such as the *Wisteria floribunda agglutinin* (WFA). PNNs regulate the intrinsic properties of the encapsulated neurons and thus aid the fast-spiking of PV-INs (Chaunsali et al., 2021). The loss of PNNs has been associated with diseases such as Alzheimer (Crapser et al., 2020) and epilepsy (Tewari et al., 2018; Chaunsali et al., 2021). Notably, WFA signals per area were drastically decreased in the hippocampus and in the somatosensory and retrosplenial cortex of 23-week-old KCC2 KO^PV^ mice (Figure 6B). The percentage of WFA-positive tdTomato-cells was also reduced in 23-week-old KCC2 KO^PV^ mice but not at 8 weeks of age (Figures 6C,D). Moreover, the WFA labeling of individual tdTomato-positive cells was reduced in 23-week-old KCC2 KO^PV^ mice indicating that the loss of PNNs precedes the loss PV-INs (Figures 6D,E).

Previously, it was described that seizures induce the expression of glial fibrillary acidic protein (GFAP) in astrocytes (Steward et al., 1992; Coulter and Steinhauser, 2015; Steinhauser et al., 2016; Siracusa et al., 2019; Sanz and Garcia-Gimeno, 2020). Indeed, GFAP immunoreactivity was strongly induced in 23-week-old KCC2 KO^PV^ mice (Figures 6F,G).

These data show profound structural changes in brains of 23-week-old KCC2 KO^PV^ mice.

### Transcriptional Profiling of PV-Positive Interneurons Upon Disruption of KCC2

**FIGURE 7:**
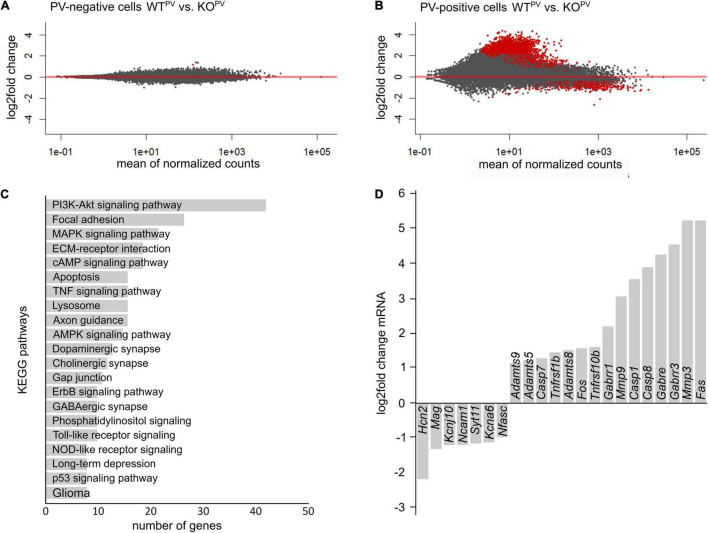
Transcriptional profiling of PV-INs. **(A,B)** MA-plot illustrating differentially expressed genes in PV-negative **(A)** and PV-positive **(B)** cells isolated from 23-week-old WT^PV^ and KCC2 KO^PV^ mice (*n* = 4/4 experiment; *N* = 8/8 mice (brains from 2 mice of the same genotype were pooled per experiment); red dots *p* < 0.05; Benjamini adjusted). **(C)** KEGG pathway enrichment analysis of 1,126 differentially expressed genes (*p* < 0.05; Benjamini adjusted). **(D)** Selected differentially expressed genes in PV-INs of KCC2 KO^PV^ mice.

To get further clues about the loss of PV-positive interneurons, we FACS sorted tdTomato-positive and negative neurons from WT^PV^ and KCC2 KO^PV^ mice at 23 weeks of age. As described recently (Pensold et al., 2020), we combined mechanical and trypsin/collagenase-based enzymatic dissociation of brain tissue with *Percoll* density gradient centrifugation. The transcriptome of tdTomato-negative neurons isolated from 23-week-old control and KCC2 KO^PV^ mice did not show major differences in the KEGG pathway term enrichment analysis as well as *Panther - Gene List Analysis* (Figure 7A).

Comparison of the transcriptome of tdTomato-positive interneurons isolated from 23-week-old control and KCC2 KO^PV^ mice revealed major changes in genes involved in PI3K-Akt signaling and MAPK signaling (Figures 7B,C), some of which have been linked with epilepsy previously (Berdichevsky et al., 2013; Pernice et al., 2016; Carter et al., 2017). Several transcripts of apoptosis related genes including the death receptors FAS and Tnfrsf10b (tumor necrosis factor receptor superfamily, member 10b) and caspases were upregulated in KCC2 KO^PV^ mice, suggesting that the loss of PV-INs is preceded by activation of pro-apoptotic pathways. We also found differences for transcripts of cell-cell contact and ECM-related genes in PV-INs isolated from KCC2 KO^PV^ mice. As reported previously following status epilepticus (Dubey et al., 2017), transcripts of matrix metalloproteinases such as *MMP3*, *ADAMTS5*, *ADAMTS8*, and *ADAMTS9* were induced (Figure 7D).

## Materials and Methods

### Animals

Mice were maintained on a C57BL/6 background and were housed in plastic cages at a 12 h day-night rhythm with constant temperature and humidity. Animals had access to food and water *ad libitum*. The body weight of the mice was measured weekly between 4 and 25 weeks of age. Hind limb spreading was scored at 4, 8, and 23 weeks of age. All animal experiments were approved by the Thüringer Landesamt für Lebensmittelsicherheit und Verbraucherschutz (TLLV). Two different mouse lines were used in the following experiments: PV-KCC2 and PV-tomato-KCC2.

Cell-type specific knockouts were obtained by mating a floxed KCC2 line reported previously (Seja et al., 2012) with PV-Cre mice (B6;129P2-Pvalbtm1(cre)Arbr/J) (Hippenmeyer et al., 2005). For labeling of PV-positive interneurons these mice were mated with the B6.Cg-Gt(ROSA)26Sortm14(CAG-tdTomato)Hze/J line (Madisen et al., 2010). All breeders were homozygous for tdTomato. Male heterozygous floxed Cre-negative KCC2 mice were mated with heterozygous floxed Cre-positive female KCC2 mice. For KCC2 genotyping we used a forward primer 5′-TCT GCC TGG AAC ACT CTC CTG C-3′ and reverse primer 5′-CAA CCT GAA CTC CCA AGG ATA CCC-3′ to amplify a 420 bp WT allele and a 465 bp *floxed* allele. For PV-Cre genotyping the primers 5′-AACAGCAGCGAGCCCGAGTAGTG-3′; 5′-TAA GAA CTA GAC CCA GGG TAC AAT G-3′; 5′-AAA CGT TGA TGC CGG TGA ACG TGC-3′ and 5′-TAA CAT TCT CCC ACC GTC AGT ACG-3′ were used to amplify a 388 bp WT allele and a 214 bp Cre allele. For tdTomato genotyping we used the WT primer 5′-AAG GGA GCT GCA GTG GAG TA-3′ and 5′- CCG AAA ATC TGT GGG AAG TC-3′ to amplify a 297 bp WT allele and the *knock-in* primers 5′-GGC ATT AAA GCA GCG TAT CC-3′ and 5′-CTG TTC CTG TAC GGC ATG G-3′ to amplify a 196 bp fragment.

### Immunofluorescence

Brains were dissected after transcardial perfusion with ice-cold 4% PFA/PBS and post-fixed in 4%PFA/PBS overnight. The tissue was subsequently immersed in 15% sucrose/PBS and 30% sucrose/PBS for cryoprotection. Brain tissue was cut into free-floating 30 μm thick sections with a sliding microtome (Leica). Sections were rinsed in PBS. 0.25% (v/v) Triton X-100 in 1 × PBS was used to permeabilize cells. After blocking with NGS (normal Goat serum; Vector Laboratories), the sections were incubated overnight at 4°C with the following primary antibodies: rabbit anti-KCC2 (Millipore, 07-432) 1:1,000, rabbit anti-Parvalbumin (Svant, PV25) 1:1,000, mouse anti-Parvalbumin (Svant, PV235) 1:2,000, mouse anti-GFAP (Millipore MAB360) 1:500 and rabbit anti-GABA (Sigma A2052) 1:1,000. To detect PNNs, we used Flourescein labeled Wisteria Floribunda Lectin (WFA, Vector Labs, FL-1351) in a dilution of 1:200. Corresponding secondary antibodies were obtained from Invitrogen (dilution 1:2,000). Nuclei were stained with DAPI 1:10,000 (Invitrogen). Sections were mounted with Fluoromount-G (Southern Biotech). Images were acquired with a Zeiss LSM 880 Airyscan confocal microscope. Z-projections with average intensities processed with ImageJ are shown. The acquisition parameters and image processing were identical for all genotypes. 3 consecutive slices (Bregma -2.3) were analyzed for quantification of cell numbers.

### Seizure Threshold

To determine the seizure threshold, 8-week-old mice were pre-treated with 423 mg/kg lithium chloride (intraperitoneally (i.p.), Sigma, L9650). 18 h later we injected 1 mg/kg Methylscopolamine (Sigma, S8502). Seizures were induced 30 min later by intraperitoneal injection of 80 mg/kg pilocarpine (Sigma, P6503). Seizures were scored for 3 h according to a modified Racine scale: 0 (no changes, normal behavior), 1 (behavioral arrest, motionless starring, orofacial automatism), 2 (head nodding), 3 (lordotic posture, chewing), 4 (rearing), 5 (falling, loss of postural control), 6 (generalized tonic-clonic activity, complete loss of control). Surviving animals were sacrificed 4 weeks later (Racine, 1972).

### Behavioral Tests

The motor performance was tested by RotaRod analysis at 6, 8, and 12 weeks of age. The RotaRod apparatus (3375-4R; TSE Systems, Germany) consisted of a striated rod providing a good grip (diameter: 3 cm), separated in four compartments (width: 8.5 cm) and located 27.2 cm above the floor grid. Mice were placed on the rod for 30 sec without rotation followed by 120 s of low speed rotation at 4 rpm. Subsequently, the mice were tested in 3 trials for 5 min (4–40 rpm). At each trial, the latency to fall was recorded.

*Fear conditioning*: Mice were placed in a chamber (d 17 × w 17 × h 25 cm, plexiglas wall, 4 lux light, 70% ethanol, fan speed 100%) and allowed to explore the surrounding area for 180 s. A tone was applied for the following 20 s (9 kHz, volume 20%, 80 dB) paired with a foot shock (unconditioned stimulus, 0.7 mA for 2 s) applied in the last 2 s via the metal grid. After additional 60 s, mice were replaced into their home cages. After 24 h, the cued test was performed to assess the tone-shock association. Mice were placed in a differently shaped box with altered color pattern (Caro patterned wall, white floor), lighting (2 lux), odor (3% acetic acid), and fan speed (50%). Mice were allowed to explore the new area for 180 s before the tone was applied for 180 s. After additional 60 s, mice were transferred into their home cages and allowed to relax for 2 h. After 2 h, they were placed in the same context as during acquisition (Plexiglas walls, metal grid floor, lightening 4 lux, odor 70% ethanol, fan speed 100%), and observed for 180 s. Animals were video recorded throughout for automatic detection of freezing behavior by the ANY-maze software (Stoelting). For freezing detection, videos were analyzed manually. Freezing time is presented as percentage of the investigated 60 s intervals (Kamprath and Wotjak, 2004; Fanselow and Poulos, 2005; Smith et al., 2007).

### Corticosterone Levels

To minimize stress, the animals were housed in sets of two and handling of the mice was avoided10 days prior to blood collection. At the day of blood collection, the animals were placed in a quiet environment. In a separate room, mice were rapidly decapitated and trunk blood collected 2 to 3 h before the end of the light cycle, when systemic corticosterone (CORT) levels peak (Helen and Atkinson, 1997). The blood was then centrifuged at 5,000 rpm for 5 min; the serum was collected and stored at −80°C until CORT analysis was performed.

Serum CORT levels were measured using an enzyme immunoassay kit (# 900-097; Enzo Life Sciences) according to the manufacturer’s instructions. The sensitivity of the assay was 27 pg/ml; all samples were run in the same assay to avoid inter-assay variability. Sample size was 8–9 mice/group. The samples were diluted 1:40 in ELISA buffer and run in duplicates. Every plate included wells with negative controls (Blank, NSB), positive controls (Total Activity TA, Maximum binding Bo) and standards #1 - #5 (#1: 20,000 pg/mL; #2: 4,000 pg/mL; #3: 800 pg/mL; #4: 160 pg/mL; #5 32 pg/mL). The ELISA plate reader was normalized against blank wells and optical density read at 405 nm with correction at 580 nm. Average net Optical Density (OD) bound for each standard and sample was calculated by subtracting the average NSB OD from the average OD bound: Average net OD = average bound OD – average NSB OD. The binding was calculated as a percentage of the maximum binding wells (Bo): Percent bound = net OD/net Bo OD * 100. Plotting a graph with the standard absorbance value as the dependent variable (*Y*-axis) and CORT concentration as the independent variable (*X*-axis) results in a standard curve. The standard curve was fitted with the exponential regression function: *y* = a*x2 + b*x + c. Solving for *x* determines the concentration of the sample.

### Electrophysiological Analysis

#### Slice Preparation for Electrophysiology

After decapitation of mice (8-10 weeks or 23 weeks of age) the brain was quickly isolated, placed in ice-cold artificial cerebrospinal fluid (aCSF: 120 mM NaCl, 3.5 mM KCl, 1.3 mM MgSO_4_ x 7 H_2_O, 12.5 mM NaH_2_PO_4_ x H_2_O, 2.5 mM CaCl_2_ x 2 H_2_O, 10 mM Glucose, 25 mM NaHCO_3_; gassed with 5% CO_2_, 95% O_2_) and cut into horizontal slices with a vibroslicer (VT 1000S, Leica instruments) as described previously (Liebmann et al., 2009). Slices (350 μm) were stored at RT in aCSF for at least 1 h until use.

#### Field Potential Recordings

After equilibration, the slices were transferred to an interface recording chamber. Slices were allowed to adapt to recording conditions for 1 h (oxygenated aCSF, 32°C, flow 2-3 mL/min). Parallel bipolar stimulating electrodes with a tip separation of 75 μm (PI2ST30.1A3, Science Products) were placed onto glutamatergic Schaffer collaterals of the hippocampus CA3 region to stimulate CA1 pyramidal neurons. Upon stimulation (pulse duration 50 μs), field excitatory postsynaptic potentials (fEPSPs) were recorded using glass microelectrodes (2-5 MΩ, filled with aCSF) impaled into the *stratum pyramidale* or the *stratum radiatum* of the hippocampal CA1 region. Slopes of fEPSPs and amplitudes of population spikes (PS) were analyzed. Data of field potential recordings were collected with an extracellular amplifier (EXT-02, NPI), low pass filtered at 4 kHz and digitally stored with a sample frequency of 10 kHz. Data acquisition and analysis of population spike (PS) amplitudes were performed using the software Signal (Cambridge Electronic Design, United Kingdom). To determine the maximal population spike amplitude or the maximal slope of fEPSP, the stimulus intensity was gradually increased (0-50 V, 5 V increment) for each experiment (interstimulus interval 30 s). The relationship between stimulus intensity and the evoked response was fitted by a sigmoid function: *R_(i)_* = *R*_*max*_/[1 + *exp*((*i_*h*_-i*)/*i*_*c*_)], where *R*_(i)_ is the response at intensity (*i*), *R*_*max*_ is the maximal response, *i*_*h*_ is the intensity at which a half-maximal response was observed and *i*_*c*_ is the intensity required to change the response *e*-fold. After determination of the half-maximal stimulus intensity, paired-pulse stimuli were applied with interstimulus intervals of 15, 20, 30, 50, 80, 120, 180, 280, 430, 650, and 1,000 ms. To assess fEPSP-PS coupling, slopes of fEPSP recorded in the *stratum radiatum* and the amplitudes of the simultaneously recorded corresponding PS in the *stratum pyramidale* were correlated. For a comparison between genotypes, mean PS amplitudes within fEPSP slope bins of 0.5 mV/ms were calculated.

#### Patch Clamp Recordings

Recordings of miniature inhibitory postsynaptic currents (IPSCs) in pyramidal neurons of the hippocampal area CA1 were performed in a submerged recording chamber mounted on an upright microscope (BX51WI, Olympus). Slices were continuously superfused with gassed aCSF (2-3 ml/min, 32°C, pH 7.3) as described previously (Sinning et al., 2011). IPSCs were recorded at a holding potential of -70 mV for at least 5 min in aCSF. Data analysis was performed off-line, with the detection threshold levels set to 5 pA for IPSCs. Recordings were performed using a CsCl-based intracellular solution (in mM): 122 CsCl, 8 NaCl, 0.2 MgCl_2_, 10 HEPES, 2 EGTA, 2 Mg-ATP, 0.5 Na-GTP, 10 QX-314 [N-(2,6-dimethyl-phenylcarbamoyl-methyl) triethylammonium bromide], pH adjusted to 7.3 with CsOH. dl-APV (30 μM, Tocris Bioscience), CNQX disodium salt (10 μM, Tocris Bioscience) were added to the perfusate. After 5 min recording of sIPSCs, extracellular solution containing 0.5 μM tetrodotoxin was washed in and 5 min mIPSCs were recorded. The following parameters were determined: frequency, peak amplitude, time constant of decay (T), half-width, and electrical charge transfer. To investigate intrinsic properties of PV-interneurons of the CA1, action potential properties and spike frequency were recorded under current clamp conditions. Prolonged current steps (600 ms) were applied from the resting membrane potential in the range of 0 to 560 pA with 40 pA increments. Patch pipettes were filled with (in mM): 140 K-methane-sulfonate, 10 HEPES, 0.1 EGTA, 4 Mg-ATP and 0.3 Na-GTP, pH 7.3.

For testing of a depolarizing or hyperpolarizing effect of GABA, tight seals (> 4 GΩ) were established in PV-INs (Otsu et al., 2020). Therefore, pipettes (4-7 MΩ impedance) were filled with HEPES buffered aCSF. The change of the membrane potential in response to pressure application of GABA (100 μM, 100 ms, 5 psi) using the Toohey Spritzer Pressure System (Toohey Company) were recorded under current clamp conditions. The action potential half-width was determined as the duration of the action potential at the voltage halfway between threshold and the peak. The amplitude of the fast after-hyperpolarization (fAHP) was determined as the delta between minimum voltage within 2 ms after the action potential peak subtracted and the threshold voltage. The Software pClamp 10 (Molecular Devices) was used for the analysis of IPSCs and current clamp recordings.

### Dissection of Neurons and Cell Sorting

For the analysis of the transcriptome of PV-positive interneurons, animals at the age of 23 weeks were sacrificed by cervical dislocation and decapitated. The brain was quickly removed with sterile instruments and placed in Gray’s Balanced Salt Solution with glucose (GBSS/Glc: 137.93 mM NaCl, 3.66 mM KCl, 2.702 mM NaHCO_3_, 1.03 mM CaCl_2_, 0.28 mM MgSO_4_x7H_2_O, 0.84 mM Na_2_HPO_4_, 0.22 mM KH_2_PO_4_, 5.56 mM glucose; pH 7.4; sterile filtered) on ice. The cortex and the hippocampus were dissected in GBSS/Glc on ice. The tissue was cut into small pieces (1 – 2 mm^3^) with a sterile razor blade and transferred into falcons in a sterile environment. After washing (gentle shaking; wash medium 98.2 ml HBSS w/o Ca and Mg; 700 μl 1M HEPES; 1 ml Penicillin-Streptomycin; 100 μl 65% glucose) the tissue was incubated for 5 min with 0.25% Trypsin/EDTA (Thermo Fisher Scientific; 4.5 ml 0.25% Trypsin/EDTA, 50 μl Penicillin-Streptomycin, 50 μl 1M HEPES, 500 μl 50% trehalose) for 30 min at 37°C. Pre-incubated DNAse (AplliChem, 50 μl, 4 μg/μl, 600 U) was added to the mix. A washing step with 2.1 ml trypsin stop-medium (40 ml DMEM/F12, 5 ml FBS, 500 μl Penicillin-Streptomycin, 5 ml 50% trehalose) led to the inactivation of trypsin. 900 μl collagenase type 2 (Worthington, 10 mg/ml in HBSS) was added to the medium and the tissue was incubated again for 25 min at 37°C. After another washing step with warm trypsin stop-medium and 3.63 μl 0.5 M EDTA, the samples were left on ice to cool down and finally resuspended in 1.5 ml trypsin stop-medium. Sterile coated (Sigmacote^®^) Pasteur pipettes with three different opening diameters (large, medium and small) were used to homogenize the tissue (pipetted up and down max 20 times). The lysate was centrifuged at 4°C (5 min, 160 rcf) and resuspended in 4 ml wash-buffer/trehalose (36 ml HBSS w/o Ca and Mg, 280 μl 1M HEPES, 400 μl Penicillin-Streptomycin, 40 μl 65% Glucose, 4 ml 50% trehalose). After another centrifugation step (4°C, 5 min, 160 rcf), the pellet was resuspended in 4 ml 30% Percoll™ (GE Healthcare, 7.5 ml Percoll™, 15 ml PBS, 2.5 ml 50% trehalose) for a density gradient centrifugation (4°C, 13 min, 600 rcf). The supernatant and the cell waste were discarded in order to resuspend the pellet in 1.0 ml wash-buffer/trehalose. Pellets were resuspended in 1.0 ml wash-buffer/trehalose and sorted (Pensold et al., 2020).

Cell suspensions prepared from the cortical hemispheres and hippocampi of 23-week-old mice were subjected to fluorescence-activated cell sorting (FACS). Following addition of DAPI, cells were sorted using an ARIA III FACS sorter (BD Biosciences, United States) with a maximal flow rate of six. The tdTomato reporter was excited by a laser (excitation 554 nm, emission 581 nm). Living cells were sorted based on a distinctive tdTomato signal. Two populations were sorted: tdTomato-positive (+) cells and tdTomato-negative (−) cells. The different cell populations (Co-tomato^+^; Co-tomato^–^; HC-tomato^+^; HC-tomato^–^) were collected in 1xPBS with 2% FBS and centrifuged (4°C; 10 min; 1,000 g). The supernatant was discarded and the cells were lysed in 150 μl TRIzol™ Reagent (Thermo Fisher Scientific) and then frozen (−80°C) for further processing.

### RNA Isolation and Sequencing

RNA was isolated and purified using the Direct-zol™ RNA MicroPrep Kit from Zymo Research. All samples were processed according to the manufacturer’s protocol and checked for integrity by capillary gel electrophoresis (Bioanalyzer, Agilent Technologies, Inc., United States). The RNA samples were stored at −80°C.

Total RNA of cells and native controls were used in biological triplicates to perform whole transcriptome analysis. Library preparation for RNA-Seq was performed using the SMARTer^®^ Stranded Total RNA-Seq Kit v2 - Pico Input Mammalian (Takara, Cat# 634412) including rRNA-depletion and further processed according to the manufacturer’s protocol. The concentrations of final cDNA libraries were determined using the Qubit 2.0 fluorometer (Invitrogen) (average 20 ng/μl) and quality checked with a Bioanalyzer (Agilent 2100 Bioanalyzer) high sensitivity DNA assay. cDNA libraries were amplified and sequenced on an Illumina NextSeq 550 system (high-output, paired-end, read length 151 nt; San Diego, CA, United States). Overall, 24 samples were sequenced, yielding between 26.2 and 105.6 million paired-end reads per sample. One sample with only 1.6 million reads was considered to be a drop-out and retained from further analysis.

After demultiplexing, the quality of the resulting FASTQ files was monitored using FastQC (https://www.bioinformatics.babraham.ac.uk/projects/fastqc/). The reads were individually mapped to the murine reference primary assembly mm10 using STAR_2.5.0c (Dobin et al., 2013) with default parameters and using additionally the –quantMode GeneCounts. Resulting ReadsPerGene tables were processed with an in house R-pipeline and differentially expression analysis carried out based on DESeq2 v.1.16.1^1^. Genes in the PV-KCC2 RNA sequencing data were considered differentially expressed with a Benjamini-Hochberg adjusted *p* value *p* < 0.05 and a | logfc| > 0,5. Gene lists were submitted to the *Database for Annotation, Visualization and Integrated Discovery1* (DAVID) for Gene Ontology (GO) or KEGG Pathway term enrichment analysis. Results of GO enrichment analysis were visualized in a bar diagram including the respective Benjamini-Hochberg corrected p-value, the number of genes and the enrichment fold change included in a certain term. All raw read data were deposited at ArrayExpress under accession E-MATB-11147.

#### Statistics

For statistical analysis, raw data were analyzed for normal distribution with the Kolmogorov-Smirnov test or with graphical analysis using the box-plot and QQ-plot. If appropriate, we used 1-way ANOVA, 2-way ANOVA, Student’s *t* test (unpaired), Kruskal-Wallis Test, Mantel-Cox Test and Fisher’s exact test. P values of less than 0.05 were considered significant. For all data, means with SEM are shown.

#### Study Approval

All animal experiments were approved by our local institutions (TLV UKJ-17-006; 02-069/16).

## Discussion

PV-INs play a major role to control the timing of pyramidal cell activity (Pouille and Scanziani, 2001), for the generation of rhythmic activities as well as the coupling of principal cells into functional assemblies (Klausberger and Somogyi, 2008; Agetsuma et al., 2018). They receive excitatory input from principal cells and GABAergic input from local (Gulyas et al., 1996) and long-range projecting (Freund and Antal, 1988) interneurons. Our immunolabeling of brain sections dissected from 8-week-old WT^PV^ mice shows that the majority of PV-INs in the hippocampus express KCC2. This is in agreement with data obtained for adult rat brains, where somata and radially running dendrites labelled for KCC2 and PV in the *strata oriens*, *radiatum* and *lacunosum moleculare* (Gulyas et al., 2001). PV-INs in the mouse cortex also label for KCC2.

Intrinsic properties such as resting membrane potential and basic passive membrane properties including membrane resistance and capacity of PV-INs were not altered in KCC2 KO^PV^ mice. This suggests that the previous finding that KCC2 affects pyramidal cell excitability through its interaction with Task-3 potassium channels (Goutierre et al., 2019) does not apply to PV-positive interneurons. Nevertheless, KCC2 appears to have an important role for the GABA response of hippocampal PV-INs, because its pharmacological inhibition depolarized the reversal potential of GABA_A_ receptor mediated currents in mice (Otsu et al., 2020). To evaluate the polarity of synaptic potentials in PV-INs we used a similar minimally invasive approach and performed cell-attached current clamp recordings from PV-positive CA1 interneurons in acute brain slices thus avoiding major perturbations of ion gradients (Perkins, 2006; Kirmse et al., 2015; Otsu et al., 2020) and found a hyperpolarizing response in the majority of PV-INs in 8-10-week-old WT^PV^ mice. Notably, a mostly depolarizing response was reported for PV-INs in 6-week-old control mice (Otsu et al., 2020). This may suggest that the maturation of the transmembrane chloride gradient in mice extends beyond 6 weeks of age.

Although most PV-INs showed a depolarizing response in KCC2 KO^PV^ mice, a depolarizing GABA response can still result in inhibition due to membrane resistance shunting (Staley and Mody, 1992; Kirmse et al., 2015). Decreased E-S coupling and diminished paired pulse facilitation in acute brain slices, however, suggest that PV-INs are disinhibited in KCC2 KO^PV^ mice. In agreement the sIPSC frequency recorded from principal cells was increased in KCC2 KO^PV^ mice, while frequencies and amplitudes of mIPSCs upon block of spontaneous events by tetrodotoxin were independent of the genotype.

Recent studies highlighted that the activity of GABAergic interneurons is controlled by GABA-mediated synaptic inhibition, which can produce oscillatory synchrony in interneuron networks (Traub et al., 1996; Wang and Buzsáki, 1996; Bartos et al., 2007; Khazipov, 2016). Inhibitory neurons can specifically suppress the firing of other inhibitory neurons (Pfeffer et al., 2009; Pi et al., 2013). Such disinhibition can lead to the selective amplification of local processing. Thus, adding to the diversity of GABAergic interneuron classes and their differential recruitment by specific patterns of excitatory input, inhibition of inhibitory GABA neurons markedly expands the range of mechanisms by which they regulate cortical network function (Kepecs and Fishell, 2014).

It is well accepted that failure of the inhibitory restraint provided by GABAergic interneurons can underlie seizure initiation and propagation in both animal models and in humans (Trevelyan et al., 2006; Schevon et al., 2012). This may also explain, why acute blockade of GABA receptors rapidly initiates seizure activity (Treiman, 2001) and drugs that increase synaptic GABA by inhibiting GABA catabolism act as effective anticonvulsants (Sills and Rogawski, 2020). Also Dravet syndrome, which is caused by inactivating mutations of the voltage gated sodium channel Nav1.1 (Claes et al., 2001; Tran et al., 2020), likely results from dampened excitability of interneurons thus decreasing the inhibitory control of the network (Yu et al., 2006). Here, we show that disinhibiting PV-INs by disrupting KCC2, which increases the GABAergic drive, decreases the seizure susceptibility, which at first sight might be counterintuitive. Increases in interneuron activity, however, can render GABA responses of the target cells depolarizing due to chloride accumulation (Payne et al., 2003; Khazipov et al., 2004; Magloire et al., 2019). Such excitatory GABA effects due to chloride accumulation may contribute to seizure induction (Cossart et al., 2005; Palma et al., 2006; Kaila and Miles, 2010; Gonzalez et al., 2018; Klein et al., 2018) because PV-INs are able to synchronize network activity (Jiruska et al., 2013; Khazipov, 2016).

Remarkably, interneurons are particularly sensitive to seizure-related damage (Sloviter Robert, 1987; de Lanerolle et al., 1989; Cossart et al., 2001; Bartos et al., 2007). Among the most vulnerable interneurons in human temporal lobe epilepsy and related animal models are those expressing PV (Bouilleret et al., 2000). As a matter of fact, we found a time-dependent decrease in PV-INs in KCC2 KO^PV^ mice. This may further increase the excitability of the network as evidenced by the decreased E-S coupling in 23-week-old KCC2 KO^PV^ mice and thus aggravate epilepsy. Indeed, focal ablation of GABAergic interneurons can cause hyperexcitability and repetitive seizures (Drexel et al., 2017; Spampanato and Dudek, 2017).

To get further clues we analyzed the transcriptional profile of PV-INs from WT^PV^ and KCC2 KO^PV^ mice. In agreement with the progressive loss of PNNs in KCC2 KO^PV^ transcripts of several metalloproteinases were up-regulated, which contribute to the remodeling of the ECM and synaptic circuit remodeling (Ferrer-Ferrer and Dityatev, 2018). More importantly, we also identified major changes in PI3K-AKT and p38-MAPK signaling. A dysregulation of the PI3K-AKT pathway is known for several disorders of the central nervous system such as Parkinson’s disease (Khwanraj et al., 2016), ischemic brain injury (Zhang et al., 2015) as well as epilepsy (Roy et al., 2015). Although PI3K-AKT is generally known for cell survival, it can also trigger apoptosis by its ability to increase reactive oxygen species and to suppress antioxidant enzymes (Los et al., 2009).

MAPKs play a pivotal role in converting extracellular stimuli into a wide range of cellular responses, including cell growth, migration, proliferation, differentiation, and apoptosis (Wada and Penninger, 2004). In particular, p38-MAPK signaling plays a key role to balance cell survival and death in response to both extracellular and intracellular stresses in a cell context-specific and cell type-specific manner, which can eventually converge on caspase activation as key effectors of apoptosis (Devanand Sarkar et al., 2002; Porras et al., 2004). Altogether, these transcriptional changes suggest that the progressive loss of PV-INs in KCC2 KO^PV^ mice is mediated via apoptosis.

The knowledge how individual neuron subtypes are affected by epilepsy and how individual subtypes contribute to epileptogenesis is very limited. Here we show a prominent role of PV-INs. Fatal spontaneous seizures, which were incidentally observed in KCC2 KO^PV^ mice starting from roughly 8 weeks of age, likely explain the high lethality of KCC2 KO^PV^ mice. Other factors, however, may contribute as well, because PV-INs are broadly expressed and involved in different neuronal circuits. For a systematic analysis of the onset, frequency and outcome of spontaneous seizures chronic electroencephalogram recordings are desirably for future analyses. Such data will shed additional light on the role of PV-INs for the pathophysiology of seizures and are necessary, in order to better understand disease etiology and discover new targets for diagnostics and treatment.

## Data Availability Statement

The raw data supporting the conclusions of this article will be made available by the authors, without undue reservation.

## Ethics Statement

The animal study was reviewed and approved by Landesverwaltungsamt Thüringen. Written informed consent was obtained from the owners for the participation of their animals in this study.

## Author Contributions

TH, MG, RD, DP, MU, and LL performed experiments and analyzed data. CH interpreted data, initiated and supervised the study and wrote the manuscript. All authors contributed to the article and approved the submitted version.

## Conflict of Interest

The authors declare that the research was conducted in the absence of any commercial or financial relationships that could be construed as a potential conflict of interest.

## Publisher’s Note

All claims expressed in this article are solely those of the authors and do not necessarily represent those of their affiliated organizations, or those of the publisher, the editors and the reviewers. Any product that may be evaluated in this article, or claim that may be made by its manufacturer, is not guaranteed or endorsed by the publisher.

## Footnotes

1
https://bioconductor.org/packages/release/bioc/html/DESeq2.html

## Supplementary Material

Supplementary Figure 1 TdTomato-labeled cells in brain sections of PV-Cre/tdTomato mice stain for PV. **(A)** Representative images from hippocampus and S1 of a brain section of an 8-week-old PV-Cre/tdTomato mouse stained for PV (green). Scale bar 100 μm. **(B)** Representative KCC2 and DAPI staining from the CA3 region of the hippocampus of 8-week-old WT^PV^ and KO^PV^ mice. Scale bars 5 μm. **(C)** The quantification of KCC2-labeled tdTomato-positive neurons in the hippocampus of WT^PV^ and KCC2 KO^PV^ mice confirms the deletion of KCC2 in most PV-INs at 8 weeks of age. KCC2 expressing tdTomato labeled cells were counted in CA1 (WT: 80.3 ± 1.9%; KO: 21.3 ± 3.1%) and CA3 (WT: 83.6 ± 2.1%; KO: 19.5 ± 1.61%) quantification from *n* = 9 sections and *N* = 3 mice; Student’s unpaired t-test; ****p* < 0.001).

Supplementary Figure 2 Motor impairment and increased anxiety in KCC2 KO^PV^ mice. **(A)** The Rotarod analysis at 6, 8, and 12 weeks of age shows a progressive decline of motor functions in KCC2 KO^PV^ mice (*N* = 15/16 mice; 2-way ANOVA; Bonferroni’s post-test; *** *p* < 0.0001). **(B)** Cued and contextual fear conditioning test in 8-week-old WT^PV^ and KCC2 KO^PV^ mice. Freezing behavior during the test was measured as an index of fear memory. For acquisition mice were placed into a conditioning chamber and were given pairings of a tone and an electric foot-shock. After 24 h mice were either exposed to a different chamber with presentation of the auditory cue (cued test) or the same context as for acquisition (context test). Both genotypes remembered the conditioned stimulus and the context of the aversive stimulus. KCC2 KO^PV^ mice displayed an increased anxiety-like behavior (2-way ANOVA; Bonferroni post-test; **p* > 0.05; ***p* < 0.001; ****p* < 0.0001). **(C)** The serum corticosterone concentration is elevated in 8-week-old KCC2 KO^PV^ mice (*N* = 8/9 mice; unpaired Student’s *t*-test; **p* = 0.016). **(D)** Duration of each Racine level after pilocarpine injection (*N* = 6/6; 2-way ANOVA; Bonferroni post-test; ns not significant; **p* > 0.05; ***p* < 0.001). **(E)** Survival of WT^PV^ and KO^PV^ mice after pilocarpine injection (*N* = 6/6; Mantel-Cox Test; **p* = 0.0337).

Supplementary Figure 3 Disinhibition of cortical PV-INs upon disruption of KCC2. **(A,B)** Paired-pulse ratios are reduced in the motor cortex (A; *n* = 35/32 slices from *N* = 12/9 mice) and somatosensory cortex (B; *n* = 34/33 slices from *N* = 13/11 mice) of KCC2 KO^PV^ mice (2-way ANOVA repeated measure; Bonferroni post-test; **p* > 0.05; ***p* < 0.001; ****p* < 0.0001). **(C)** The frequency but not the amplitude of spontaneous inhibitory post synaptic currents (sIPSCs) is increased in the somatosensory cortex of KCC2 KO^PV^ mice (*N* = 6/6 mice; unpaired Student’s *t*-test; Kolmogorov-Smirnov test; ns not significant; **p* = 0.0137). **(D)** Miniature inhibitory postsynaptic currents (mIPSCs) upon tetrodotoxin (TTX) inhibition are not changed in 8-week-old KCC2 KO^PV^ mice (*N* = 6/6 mice; unpaired Student’s *t*-test; Kolmogorov-Smirnov test; ns not significant).

Supplementary Figure 4 Altered neurites in 23-week-old KCC2 KO^PV^ mice. TdTomato-signals from S1 (left), the retrospenial (RSP) cortex (middle), and the hippocampus of WT^PV^ (upper) and KCC2 KO^PV^ (lower) brain sections. Scale bars 100 μm.

Supplementary Table 1 Individual numbers for the quantification of tdTomato-positive cells displayed in Figures 5D–G.
