# Supplementary figures and images for: Disruption of KCC2 in Parvalbumin-Positive Interneurons Is Associated With a Decreased Seizure Threshold and a Progressive Loss of Parvalbumin-Positive Interneurons
Source: Front Mol Neurosci. 2022 Feb 3;14:807090. doi: 10.3389/fnmol.2021.807090 (PMC8850922; doi:10.3389/fnmol.2021.807090)

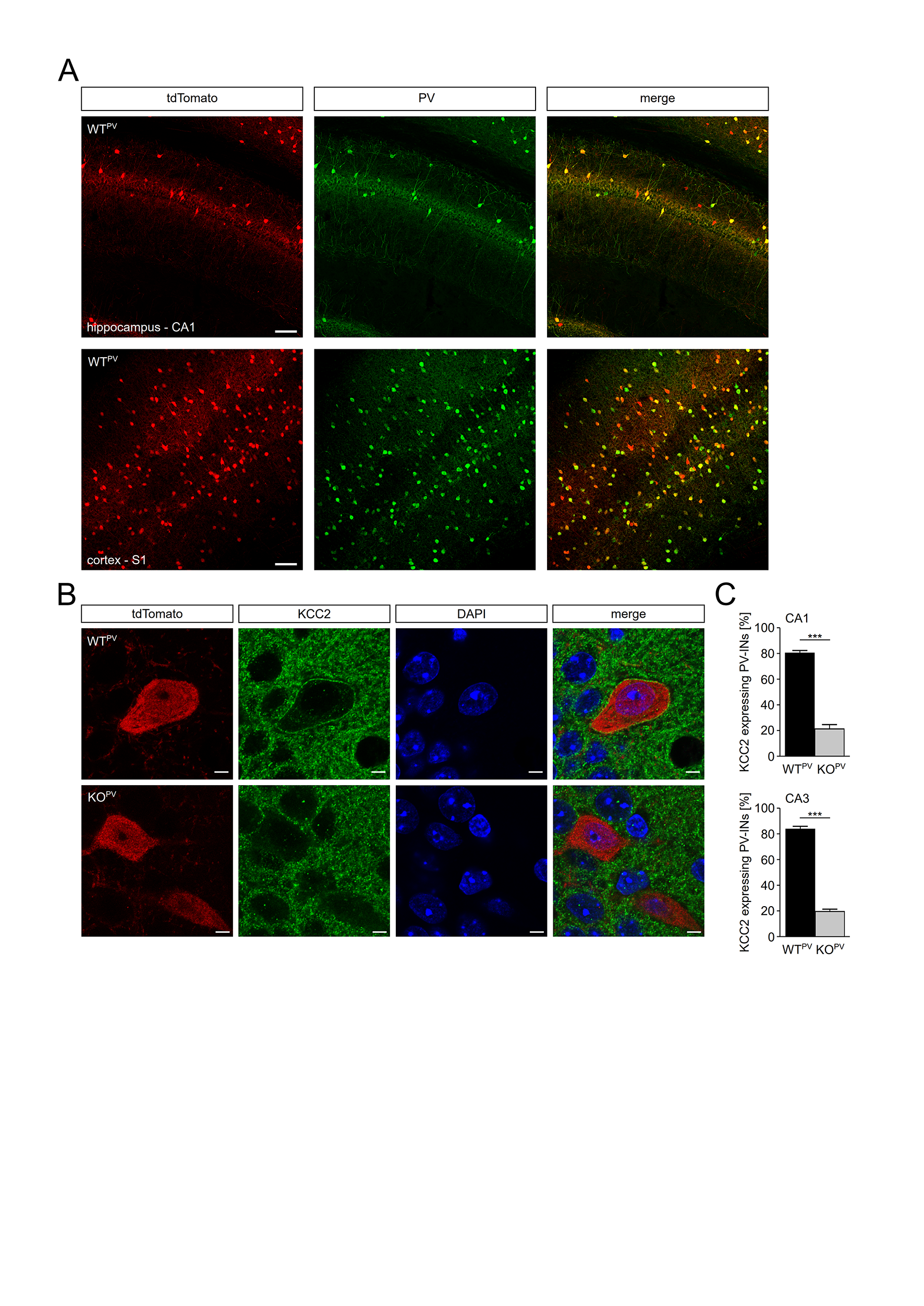

Supplement: Supplementary Figure 1 — TdTomato-labeled cells in brain sections of PV-Cre/tdTomato mice stain for PV. (A) Representative images from hippocampus and S1 of a brain section of an 8-week-old PV-Cre/tdTomato mouse stained for PV (green). Scale bar 100 μm. (B) Representative KCC2 and DAPI staining from the CA3 region of the hippocampus of 8-week-old WTPV and KOPV mice. Scale bars 5 μm. (C) The quantification of KCC2-labeled tdTomato-positive neurons in the hippocampus of WTPV and KCC2 KOPV mice confirms the deletion of KCC2 in most PV-INs at 8 weeks of age. KCC2 expressing tdTomato labeled cells were counted in CA1 (WT: 80.3 ± 1.9%; KO: 21.3 ± 3.1%) and CA3 (WT: 83.6 ± 2.1%; KO: 19.5 ± 1.61%) quantification from n = 9 sections and N = 3 mice; Student’s unpaired t-test; ***p < 0.001). [file Image_1.TIF]

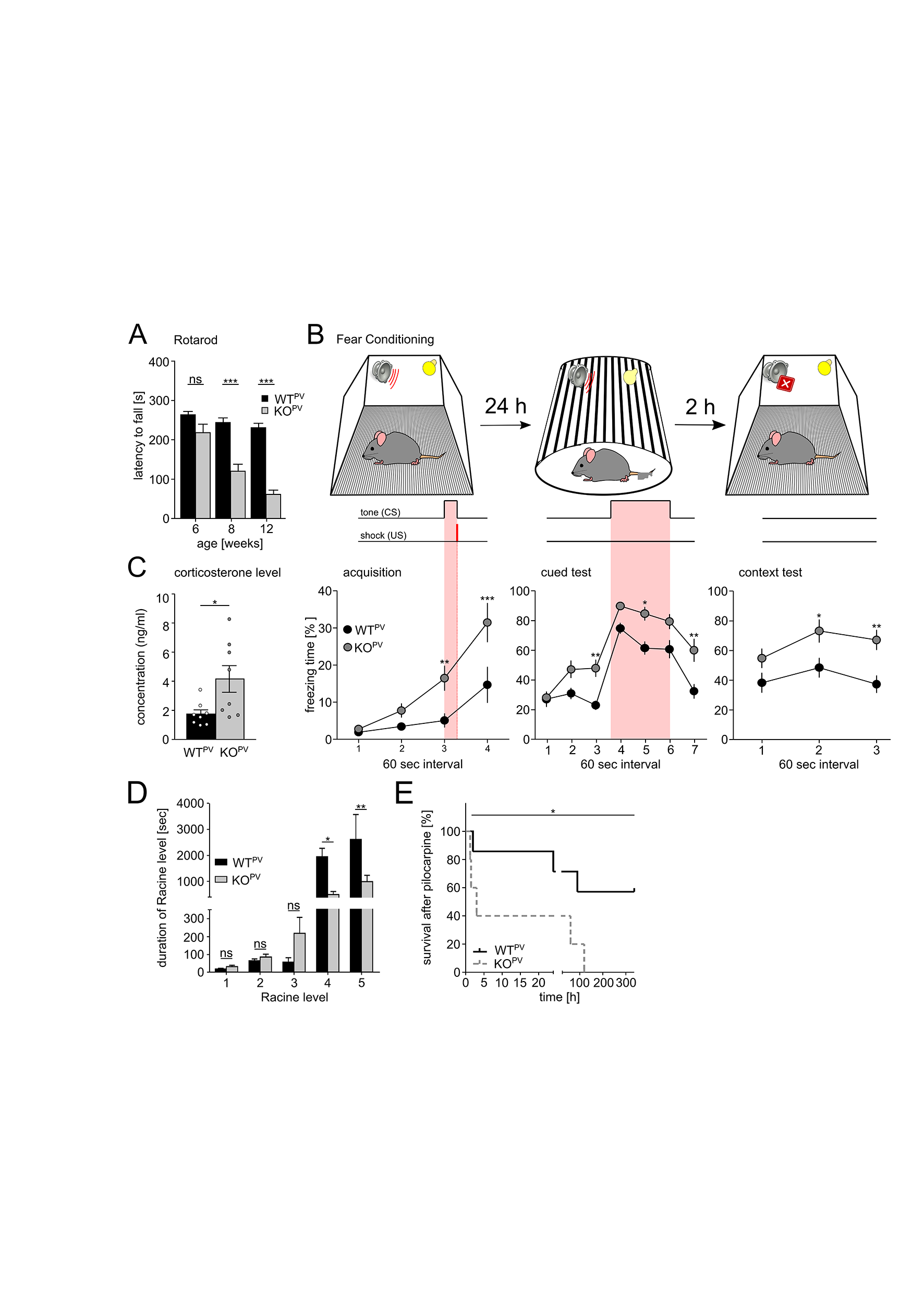

Supplement: Supplementary Figure 2 — Motor impairment and increased anxiety in KCC2 KOPV mice. (A) The Rotarod analysis at 6, 8, and 12 weeks of age shows a progressive decline of motor functions in KCC2 KOPV mice (N = 15/16 mice; 2-way ANOVA; Bonferroni’s post-test; *** p < 0.0001). (B) Cued and contextual fear conditioning test in 8-week-old WTPV and KCC2 KOPV mice. Freezing behavior during the test was measured as an index of fear memory. For acquisition mice were placed into a conditioning chamber and were given pairings of a tone and an electric foot-shock. After 24 h mice were either exposed to a different chamber with presentation of the auditory cue (cued test) or the same context as for acquisition (context test). Both genotypes remembered the conditioned stimulus and the context of the aversive stimulus. KCC2 KOPV mice displayed an increased anxiety-like behavior (2-way ANOVA; Bonferroni post-test; *p > 0.05; **p < 0.001; ***p < 0.0001). (C) The serum corticosterone concentration is elevated in 8-week-old KCC2 KOPV mice (N = 8/9 mice; unpaired Student’s t-test; *p = 0.016). (D) Duration of each Racine level after pilocarpine injection (N = 6/6; 2-way ANOVA; Bonferroni post-test; ns not significant; *p > 0.05; **p < 0.001). (E) Survival of WTPV and KOPV mice after pilocarpine injection (N = 6/6; Mantel-Cox Test; *p = 0.0337). [file Image_2.TIF]

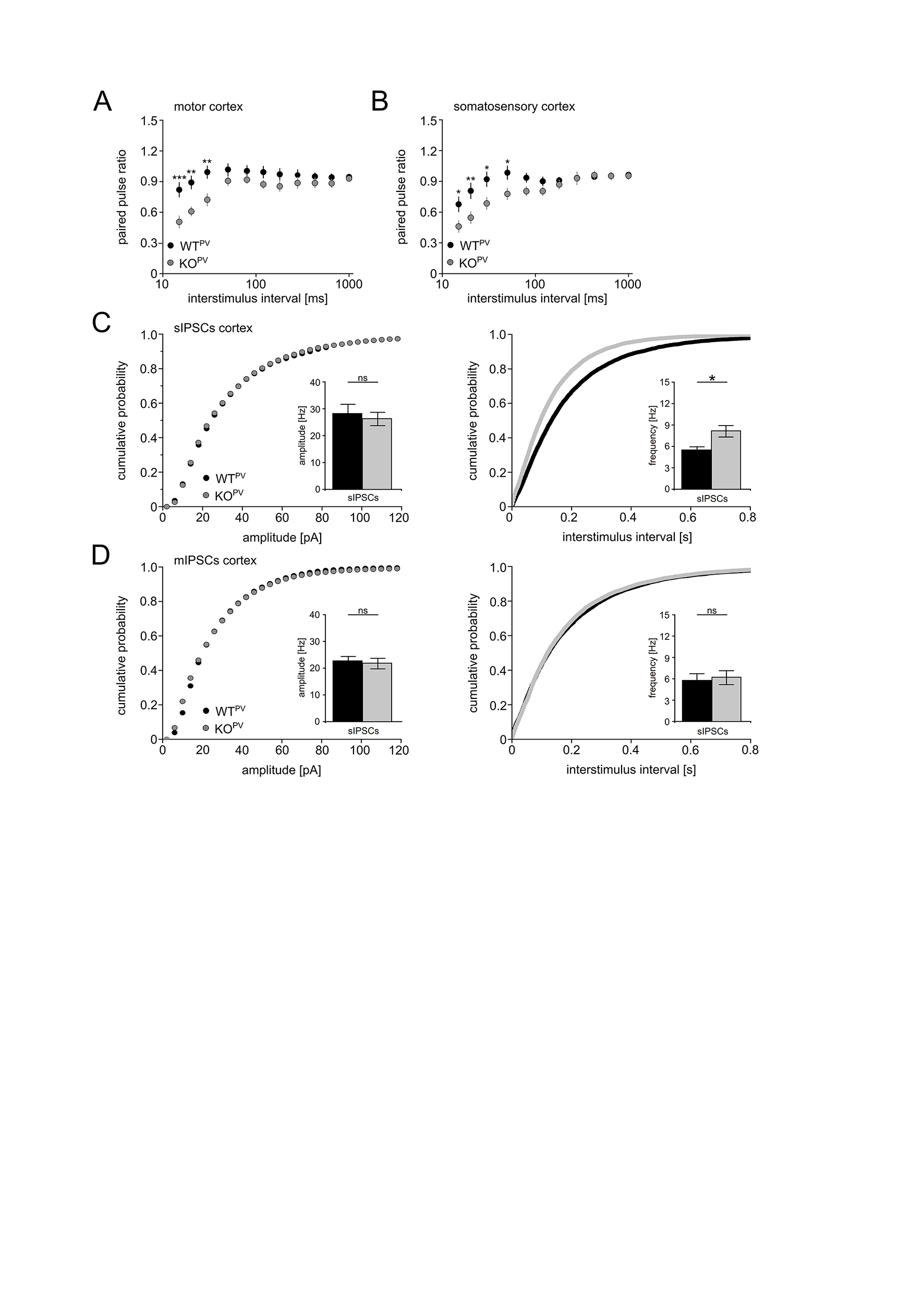

Supplement: Supplementary Figure 3 — Disinhibition of cortical PV-INs upon disruption of KCC2. (A,B) Paired-pulse ratios are reduced in the motor cortex (A; n = 35/32 slices from N = 12/9 mice) and somatosensory cortex (B; n = 34/33 slices from N = 13/11 mice) of KCC2 KOPV mice (2-way ANOVA repeated measure; Bonferroni post-test; *p > 0.05; **p < 0.001; ***p < 0.0001). (C) The frequency but not the amplitude of spontaneous inhibitory post synaptic currents (sIPSCs) is increased in the somatosensory cortex of KCC2 KOPV mice (N = 6/6 mice; unpaired Student’s t-test; Kolmogorov-Smirnov test; ns not significant; *p = 0.0137). (D) Miniature inhibitory postsynaptic currents (mIPSCs) upon tetrodotoxin (TTX) inhibition are not changed in 8-week-old KCC2 KOPV mice (N = 6/6 mice; unpaired Student’s t-test; Kolmogorov-Smirnov test; ns not significant). [file Image_3.TIF]

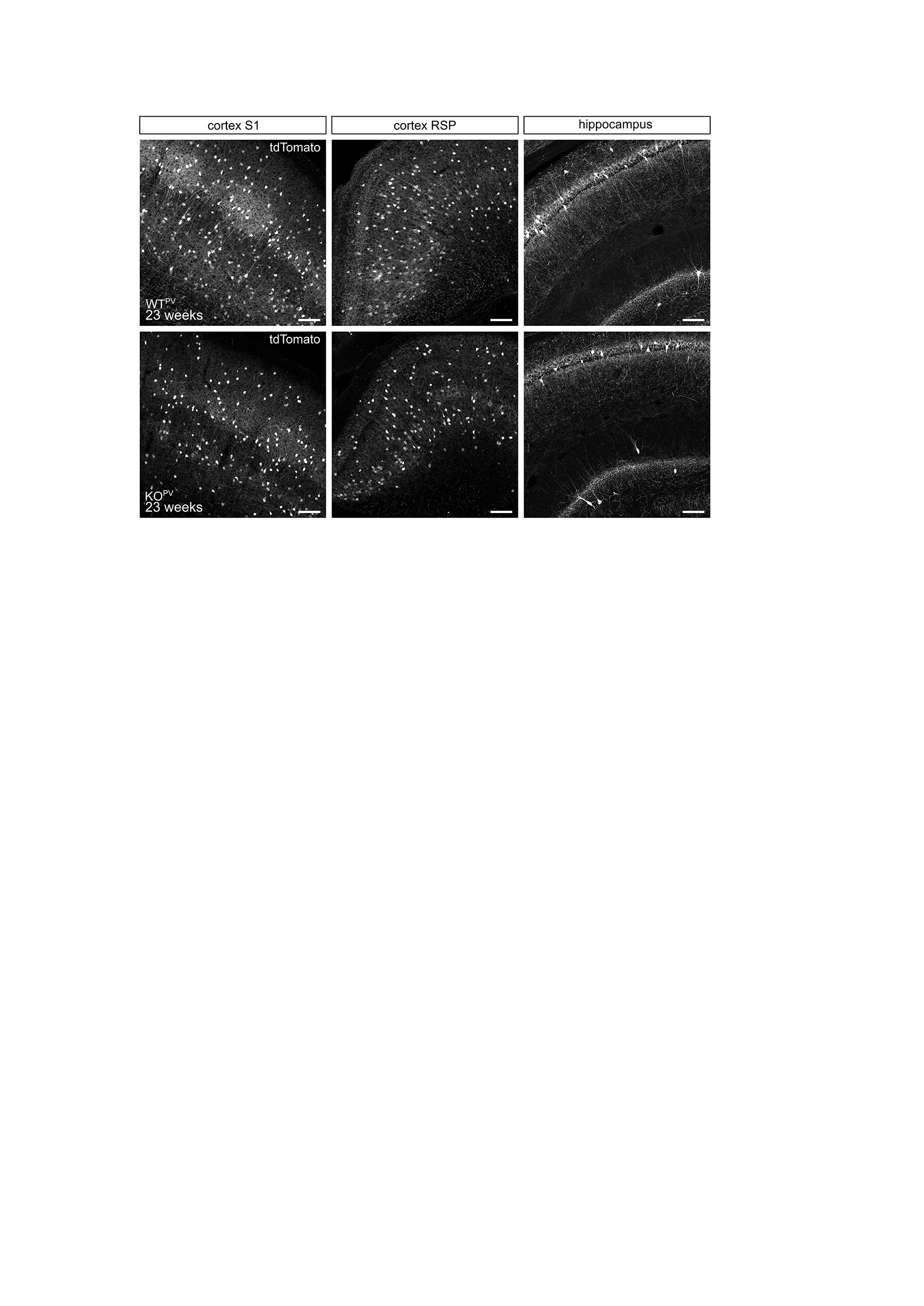

Supplement: Supplementary Figure 4 — Altered neurites in 23-week-old KCC2 KOPV mice. TdTomato-signals from S1 (left), the retrospenial (RSP) cortex (middle), and the hippocampus of WTPV (upper) and KCC2 KOPV (lower) brain sections. Scale bars 100 μm. [file Image_4.TIF]
