# Supplementary material for: Disruption of KCC2 in Parvalbumin-Positive Interneurons Is Associated With a Decreased Seizure Threshold and a Progressive Loss of Parvalbumin-Positive Interneurons
Source: Front Mol Neurosci. 2022 Feb 3;14:807090. doi: 10.3389/fnmol.2021.807090 (PMC8850922; doi:10.3389/fnmol.2021.807090)
Supplement: Supplementary Table 1 — Individual numbers for the quantification of tdTomato-positive cells displayed in Figures 5D–G. [file Table_1.DOCX]

|  | **WT 8 weeks** | | **KO 8 weeks** | | **WT 23 weeks** | | **KO 32weeks** | |
| --- | --- | --- | --- | --- | --- | --- | --- | --- |
|  | mean | SEM | mean | SEM | mean | SEM | mean | SEM |
| **hippocampus** | 102.8 | 3.1 | 92.0 | 2.7 | 105.5 | 2.6 | 73.7 | 2.8 |
| **cortex S1** | 26.6 | 0.7 | 25.9 | 0.7 | 27.0 | 1.1 | 22.8 | 0.4 |
| **cortex RSP** | 27.3 | 4.2 | 24.7 | 4.9 | 28.5 | 6.4 | 22.8 | 3.6 |
| **cortex layers** |  |  |  |  |  |  |  |  |
| II-III | 15.0 | 0.8 | 15.1 | 0.6 | 16.8 | 0.8 | 16.5 | 0.7 |
| IV | 42.4 | 1.5 | 39.9 | 1.7 | 41.0 | 2.3 | 34.4 | 1.3 |
| V-VI | 36.9 | 1.5 | 32.4 | 1.4 | 36.5 | 1.6 | 26.4 | 0.7 |
